# The Fraternal Relations of Physicians1Read before the Medical Society of Jefferson County, N. Y., January 12, 1897.

**Published:** 1897-04

**Authors:** J. R. Sturtevant

**Affiliations:** Theresa, N. Y.


### THE FRATERNAL RELATIONS OF PHYSICIANS.[1]

By J. R. STURTEVANT, M. D., Theresa, N. Y.

IN this age of fraternal organisation the world is overrun with societies created from real or imaginary necessity, most of which have, at least ostensibly, the mutual welfare and happiness of its members. Some of them are bound together by iron-clad obligations, and most of them are required to promise, with more or less emphasis, in the way of theoretical penalty, to stand by each other in trouble and to defend each other when wrongfully persecuted and to relieve one another in distress. The personnel of many of these organisations is made up of individuals from all the walks and occupations in life. The Masons, Odd-Fellows, Knights of Pythias, and the like, are men of both humble and prominent station. They are men or women in various occupations, and yet they are bound together in one common band or brotherhood for the general good of the order to which they belong and the particular good of the individual member. Others are composed of people in certain occupations and confined to such; others to certain general classes, such as mechanics, manufacturers, and the like, all of whom are supposed, by their regulations, to contribute, to the best of their abilities, to the general good of their respective organisations. Most of these are secret societies, and their doings are shrouded in more or less mystery; and there is not one such organisation but to which a large part of the world is perfectly indifferent, because they are organised in the interest of a certain class or a certain chosen few.

The world looks to none of them in the moments of greatest physical peril. Secret society relations rarely count when the life-blood is ebbing away, or when disease has fastened its grip upon the victim. It is to another class that the whole world rushes madly headlong for succor and relief from the perils that do not have commercial value. We who are gathered here today belong to this class. Our calling is the noblest, most far-reaching in its

---

1. Read before the Medical Society of Jefferson County, N. Y., January 12, 1897.

benefits, the most in demand of any occupation upon the earth. Those especially of us who have, for a goodly number of years, borne the trials, the anxieties, the privations of many kinds, that we might relieve human suffering, bring joy to the anxious household whose loved ones have tottered on the verge of eternity and are being nursed back to life, appreciate, to some degree at least, the true friendship, self-sacrificing love and devotion of those of our fellows who have shared our responsibilities and professional anxieties, and who have even deserted all personal and private interests and hastened to the rescue and relief of our own loved ones, sparing themselves no fatigue, privation or expense, forgetting self entirely in their eagerness to bring happiness and health to us and ours.

Who of us who have had loved ones' lives imperiled by disease or injury to that extent that our personal and filial anxieties disable our judgment, have not felt that thrill of gratitude to, and love for, that medical friend who, as our loved family physician, hastens to us, shifts the burden of responsibility onto his own broad and willing shoulders, and taking the helm, guides our craft past the dangerous shoals and into the quiet haven of recovery ?

While among the laity we find many noble and appreciative examples of manhood and womanhood who are mindful of the feelings of their medical adviser and are duly appreciative of his efforts to relieve their ills, you and I know too well that these are in the minority, and of them it may be said that

> "God and the doctor they alike adore,
> But only when in trouble, not before ;
> The danger o'er, both are alike requited—
> God is forgotten and the doctor slighted."

There is a large class who hold the doctor to be a common target for criticism and curses in return for his best endeavors to do them good and make them happy. This we have learned to expect, and the "older ducks have oiled their feathers" accordingly. But there is another persecution to which we are too often subjected—namely, at the hands, or rather at the tongues, of members of our own profession. We cannot deny that, like churches and societies, we have members unworthy the name ; but I do claim that we too often forget that life is too short to spend in criticising and endeavoring to build ourselves a habitation by trying to pull down that of our colaborers. What is still more deplorable and lamentable is the spectacle of a medical man lending his aid to the injury of a member of his own profession, who, struggling hard to attain well-earned success, has, perhaps, through the avarice of some unprincipled layman, been dragged into court upon the pretense of having inflicted injury to one whom he has struggled hard to restore to health and physical prosperity. How sad, and yet how disgusting, to see members of our profession, for a mere pecuniary temptation, ascend the witness stand, after hours of consultation with counsel, and endeavor to so distort the true theory of a case as to bring calumny and financial ruin upon one whose professional reputation and attainments the witness knows to be beyond reproach. With what gusto we sometimes see such witness preface his testimony with a recounting of his own reputation, the honors which may, either deservedly or undeservedly, have been heaped upon him; others, perhaps younger, recounting their own supposed skill and referring to a reputation which they have yet to earn, display their skill in building up hypotheses at the possible expense of a brother far more worthy and skilful than themselves. And all this, not even because they may have some personal debt of revenge to pay, which is said to be merely human, but for the paltry sum of an expert witness fee. To obtain that, they are willing that the professional life-blood of a worthy brother physician should freely flow, they themselves possibly being the assassins instead of the one who primarily seeks the prosecution.

Thank God! better times are coming—aye, they are at hand; and I am proud of the physicians of Jefferson county, who almost to a man rise above this nefarious sort of business, and I am proud, too, of those noble specimens of the profession from other counties who are ever ready to sacrifice their immediate private gain that truth and justice may prevail. I am proud of the spirit which animated a medical friend, who, when asked to loan his books to the prosecution in a certain case, promptly referred the limb of the law to a certain tropical clime, the only place where literature worthy of such a cause could be found.

I do not advocate the defense of crime or criminal ignorance, but I do denounce, in unmeasured terms, the act of that physician who will ascend the witness stand for the prosecution of a physician simply because he is hired to do so, of which we have had several examples in the not far distant past. If poverty is his excuse, let him remember that there is a poverty more serious than that of the purse—a poverty of manhood, of honor, of truth and justice, as applied to the efforts of his own hard-worked and industrious contemporaries, is usually associated with a poverty of good common sense. A recapitulation of the number of societies and associations in which one may have held membership or office will not count in the day of judgment when a man's fellow-laborers size him up and stamp upon his name its real value. Clay models, never so skilfully formed, will not take the place of that other gray matter, never so fearfully wanting, and which is so essential to the building up of a desirable reputation. Our legal friends are not supposed to be especially interested in the preservation of harmony in other professions than their own, and the tempting morsels of flattery by which they would beguile the unwary doctor into the witness box should be shunned and the noble example of Him who, upon the mountain top, taught how to avoid temptation, should be imitated, and our Blackstonian friends directed to greener pastures. The petty and puerile jealousies which are too prevalent in our own ranks are unworthy of a place in the program of our busy lives, and should never be a factor in determining our course when stern duty calls upon us to stand for the right. This short life is too brief to be thus wasted. The noblest examples in our profession are those who love their chosen calling for the good which it is possible for them to accomplish, who are never heard to speak ill of another member of it, who scorn to be made tools of to be used against worthy and skilful colaborers, and whose hearts throb in true sympathy for those who, like themselves, are giving the best there is in their lives for suffering mankind.

It may be safely stated that, invariably, time hangs heavily upon the hands of him who poses as a chronic expert witness against those of his own profession; and he can advertise that melancholy fact in no more certain manner. A charitable institution for the care and sustenance of this class of unfortunates, although perhaps unique, might remove them from temptation and care for them, not only when in a senile condition, but in that other condition of earlier professional career where nature has failed to supply those noble traits of character so essentially necessary in the deservedly successful physician and surgeon.

It has been suggested that an effort be made to introduce into the statutes of our own state, the New Hampshire law, which provides that when a physician or surgeon is sued for malpractice, the plaintiff must first give bonds in the sum of one-fourth the amount of the damages claimed, to be paid to the defendant in case of a verdict for the latter. This provision would no doubt discourage many blackmailers, but, with or without such legislation, we need a more keen sense of professional honor among certain medical men.

The would-be expert witness against his fellow, crams for hours or days it may be, that he may do that brother a wrong, and pocket a few ill-gotten dollars. He may better spend that precious time in cramming for the general good and for the replenishing of his own limited store of professional attainments.

The world expects and demands miraculous results at the hands of the physician. It is willing to grant him nothing, and seeks to punish him for failing to accomplish the impossible. The legal fraternity, whose professional errors and careless oversights go unpunished, and are usually committed at the expense of their clients, are not slow to avail themselves of the aid of brother against brother. Who, then, will be for us if these are against us? There are no holier or more sacred ties outside the family circle than those which should bind us together as physicians. No one else can give us that sympathy which we so much need during the fearful ordeals of responsibility which are constantly in our pathways, and why should we do otherwise than lend that sympathy and love which inspires more than all else to nobler aspirations and more effectual work for the sick and injured.

